# Placental Chorangiocarcinoma a Specific Histological Pattern of Uncertain Incidence and Clinical Impact: Systematic Review of the Literature

**DOI:** 10.3390/jcm12093065

**Published:** 2023-04-23

**Authors:** Guglielmo Stabile, Maria Sole Scalia, Tamara Stampalija, Matteo Bruno, Antonio Simone Laganà, Andrea Sartore, Alessandro Mangogna, Stefania Carlucci

**Affiliations:** 1 Institute for Maternal and Child Health, IRCCS “Burlo Garofolo”, Via dell’Istria 65/1, 34137 Trieste, Italy; 2 Department of Medicine, Surgery and Health Sciences, University of Trieste, 34137 Trieste, Italy; 3 Unit of Obstetrics and Gynecology, San Salvatore Hospital, 67100 L’Aquila, Italy; 4 Unit of Gynecologic Oncology, ARNAS “Civico—Di Cristina—Benfratelli”, Department of Health Promotion, Mother and Child Care, Internal Medicine and Medical Specialties (PROMISE), University of Palermo, 90127 Palermo, Italy

**Keywords:** chorangiocarcinoma, placenta, throphoblast proliferation, chorangioma

## Abstract

Chorangiocarcinoma is a very rare and misdiagnosed placental neoplasm. The unique morphologic features of the lesion distinguish it from other trophoblastic tumors and vascular abnormalities. We present a systematic review of the literature to provide clarity on chorangiocarcinoma entity and biology. A literature search was carried out in December 2022 using the keywords “Placental chorangiocarcinoma”, “Chorangioma”, “Placenta”, and “Throphoblast proliferation”. Articles published from 1988 to 2022 were obtained from Scopus, Google Scholar, and PUBMED. In our review, we examined maternal age, gestational age at the time of delivery, parity, type of pregnancy, placental weight, ultrasound features of the placenta, macroscopic examination and tumor size, microscopic examination, immunostaining, maternal beta-human chorionic gonadotropin, fetal and maternal outcome. Eight manuscripts were detected. They are all case reports. The macroscopic characteristics of the lesions were represented by the presence of a grey-yellow-white color well-demarcated round nodule. Microscopically, all the authors described typical aspects of malignancy as a high rate of mitosis, nuclear atypia and necrotic areas. In some cases, the presence of AE1/AE3 cytoplasmic positivity, p63 nuclear staining, and beta-human chorionic gonadotropin (BHCG) were reported. A good fetal outcome was reported in all cases of newborns with normal birth weight, except one with fetal growth restriction. Maternal outcome was good in all cases except one with maternal lung metastasis three months after delivery. The clinical course has probably underestimated the real incidence of the pathology. Only greater knowledge of its histology and its clinical course will allow us to evaluate the real prevalence of the disease.

## 1. Introduction

According to the classification of placental lesions, fetal stromal–vascular lesions encompass a subgroup of developmental conditions such as delayed villous maturation, villous capillary lesions (chorangioma, chorangiosis, chorangiomatosis, multifocal choangiomatosis), and dysmorphic villi (resembling features seen in aneuploid gestations), which can impact negatively on the mother and her fetus during pregnancy [1,2,3,4]. On the other hand, trophoblastic tumors encompass benign or pre-malignant tumors (such as hydatidiform mole, placental site nodule, placental site exaggeration), and malignant lesions (such as choriocarcinoma, placental site tumor, invasive mole) [3].

Chorangiocarcinoma is a very rare and misdiagnosed [5] placental neoplasm, of which only seven cases are described in the literature. The unique morphologic features of the lesion distinguish it from other trophoblastic tumors and vascular abnormalities, and it seems to be characterized by an abnormal trophoblastic proliferation associated with a hypervascular chorangiosis in the stroma of chorionic villi. The etiology and pathogenesis of the lesion are still undetermined. While the pathogenesis of known villous capillary lesions is related to an over-expression of vascular growth factors due to a chronic hypoxic insult [6,7,8], there is no difference between the expression level of angiogenic factors (vascular endothelial growth factor, basic fibroblast growth factor, Ang-1, and Ang-2, and platelet-derived growth factor) in chorangiocarcinoma and normal villi. Only one case reported the anomaly in one placenta of a twin dichorionic pregnancy [9].

Herein, we present a systematic review of the literature to provide clarity on chorangiocarcinoma entity and biology. Secondarily, we want to prove the hypothesis brought by Khong [5] for whom chorangiocarcinoma incidence is higher than given by the few case reports implied by the literature, and this is because of its diagnostic difficulty.

## 2. Materials and Methods

This research was approved by the Institutional Review Board of the IRCCS Burlo Garofolo, Trieste, Italy. (RC 08/2022).

**Figure 1:**
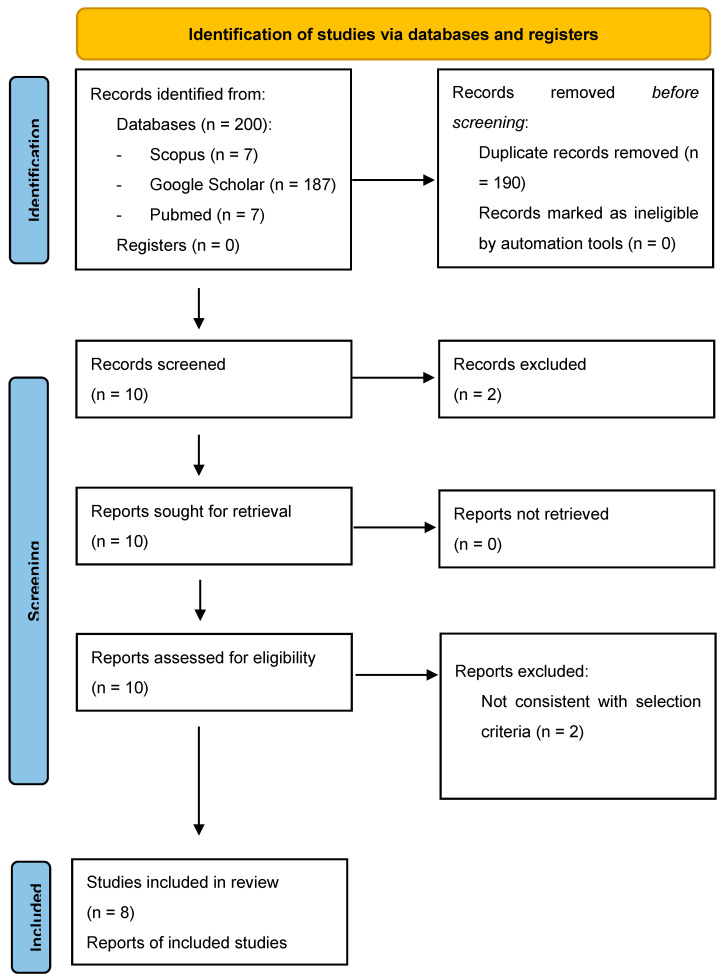
PRISMA flow Diagram.

A literature search was carried out in December 2022 using the keywords “Placental chorangiocarcinoma”, “Chorangioma”, “Placenta”, and “Throphoblast proliferation”. Articles published from 1988 to 2022 were obtained from Scopus, Google Scholar, and PUBMED. In our review, we examined maternal age, gestational age at the time of delivery, parity, type of pregnancy (singleton/twin), placental weight, ultrasound features of the placenta, macroscopic examination and tumor size, microscopic examination, immunostaining, maternal beta-human chorionic gonadotropin (basal and at follow up), fetal and maternal outcome, and pathology follow up. We excluded from the review all papers that involved cases of single chorangioma or single trophoblastic proliferation. Articles not relevant to the topic were also excluded. All studies identified were examined for their year, citation, title, authors, abstract, and full texts. Duplicates were identified through manual screening performed by two researchers and then removed. PRISMA guidelines were followed [10]. The PRISMA flow diagram of the selection process is provided in Figure 1. The systematic review was not submitted to Prospero [11] as only a limited number of case reports were found in the literature. Three authors independently screened titles and abstracts of all non-duplicated papers and excluded those not pertinent to the topic. The same three authors independently reviewed the full text of papers that passed the first screening and identified those to be included in the review. Disagreements were resolved by finding a consensus among researchers. Due to the rarity of this pathology, the studies included are all case reports. For this reason, we present the data in a descriptive manner. The inclusion of only case reports in this review presents a risk of bias. We used the Joanna Briggs Institute (JBI) Critical Appraisal tool checklist [12] for case reports to assess the methodological quality of the included studies (Table 1).

## 3. Results

We identified 200 manuscripts. Records identified through databases searching were 7 from Pubmed, 7 from Scopus, 187 from Google Scholar. Records excluded for selection criteria and duplicates were *n* = 190. We found ten cases of chorangiocarcinoma published. A case series was excluded because showing incomplete clinical data (pathological data). Consequently, there were left eight more cases (see Figure 1). They were all case reports [Table 2]. In our analysis, the median age of women affected by the pathology was 32 years, the median gestational age at the delivery was 36.4 weeks, and in 75% of the cases, the women had at least a previous pregnancy. The pregnancies were mostly singleton except for two that were multiple dichorionic–diamniotic. The average placental weight was 559.8 g. The suspicion of placental pathology at ultrasound evaluation was raised only in 3 out of 8 cases, and the lesions were described as hyperechogenic with hypoechogenic areas with or without hypervascularization. The mean major diameter of the placental lesions was 4.3 cm. The macroscopic characteristics of the lesions were represented by the presence of a grey-yellow-white color well-demarcated round nodule. Microscopically, all the authors described typical aspects of malignancy as a high rate of mitosis, nuclear atypia, and necrotic areas. In some cases, the presence of AE1/AE3 cytoplasmic positivity, p63 nuclear staining, beta human chorionic gonadotropin (BHCG), PLAP, and Ki-67 positivity were reported. Maternal BHCG decreased rapidly after delivery, being negative 1 month after delivery in the majority of cases. Only in one case, there was an increase of the BHCG at 6 weeks after delivery, which ended in lung metastasis 3 months after delivery. Good fetal outcome was reported in all cases of newborns with normal birth weight, except one who had fetal growth restriction. Patient follow-up was reported in 6 out of 8 cases and was performed from a minimum of 1 month to a maximum of 9 months after delivery. The maternal outcome was good in all cases except the case reported by Huang et al. [15] with maternal lung metastasis three months after delivery. Subsequent oncological follow-up was not systematically reported.

## 4. Discussion

Chorangiocarcinoma is a controversial proliferation of uncertain nosology. It has been described as a variant of chorangioma characterized by a high proliferative index featuring an admixture of syncytiotrophoblast and cytotrophoblast with nuclear atypia [18]. Jauniaux and colleagues [13] introduced the term “chorangiocarcinoma” to suggest that this tumor was the “missing link” between chorangiomas and choriocarcinomas. Chorangiocarcinoma is an exceedingly rare placental tumor likely of trophoblastic lineage, with few cases that have been published in the pertinent literature [Figure 1].

However, it has been argued that its frequency is likely higher than what is reported [4]. In fact, several reports emphasized the frequency of associated trophoblast hyperplasia in chorangiomas [5,19]. Khong et al. [4] described that hyperplasia was present in 50–65% of the cases, as corroborated by an increased proliferative index ranging between 50 and 65% together with an increased MIB-1 (Ki-67) staining. This finding may be related to excessive amounts of growth factors. The Ki-67 is a non-histone nuclear protein expressed throughout the active phase of the cell cycle and is a marker of cell proliferation.

As regards trophoblastic proliferation, chorangiomas with significant atypia have been called “atypical chorangiomas” or “chorangiomas with trophoblastic proliferation” [5,20,21,22]. On the other hand, chorangiomas with exuberant proliferating nodules of pleomorphic, atypical trophoblast associated with necrosis (unequivocal malignant trophoblastic component), closely related to choriocarcinoma, with or without metastases, should be defined chorangiocarcinoma [13,14,15].

Chorangiocarcinoma is usually an incidental finding in a term or a near-term placenta. Grossly, the macroscopic characteristics of the lesions are represented by the presence of well-demarcated round nodules of grey-yellow-white color and the presence of necrosis. The mean major diameter of the placental lesions was 4.86 cm.

Microscopically, it is characterized by an abnormal trophoblastic proliferation associated with hypervascular chorangiosis (or chorangioma) in the stroma of chorionic villi [23,24,25,26]. The cells in the epithelial compartment form solid masses with massive central coagulative necrosis, which is surrounded by a few (three to six) layers of viable epithelial (trophoblastic) tumor cells. At low magnification, the necrotic areas may be predominant. The epithelial pleomorphic cells have prominent nucleoli and frequent mitotic figures. The viable tumor cells in the epithelial component are positive for BHCG. The percentage of MIB-1 (Ki-67) labeled epithelial cells is high (>90%) in the viable epithelial. In contrast, the vascular/chorangiosis component (angiomatous part of the tumor) is negative for cytokeratin and HSD3B1 but is positive for vimentin, CD31, CD34, and factor 8 [14,27]. The presence of AE1/AE3 cytoplasmic positivity, p63 nuclear staining, and PLAP were reported in some cases.

The pathogenesis in the development of chorangiocarcinoma is undetermined. It has been proposed that the lesion may represent either a chorangioma with associated trophoblastic hyperplasia or a true trophoblastic neoplasm with reactive chorangiosis. It has also been speculated that these lesions might reflect a reactive proliferation of trophoblastic cells and villous vascular channels or a collision tumor of chorangioma and choriocarcinoma [7,15,25]. Considering the histological and cytological complexity of this type of tumor and its rarity, one can understand the difficulty of gynecologists and even pathologists in its diagnosis.

The clinical expertise with chorangiocarcinoma is very limited. However, reviewing the cases available in the literature, we tried to define similarities among the cases in order to delineate a unique pattern of recognition. In terms of maternal age, no one seems to be more exposed than the others (mean maternal age: 32 years). The first characteristic that recurs in the selected clinical cases is the prematurity of five over eight of them. Six over eight underwent cesarean section. Although the bias given by the small number of cases and that one and two out of 6 c-sections, respectively, were elective or had as indication vulvar condylomatosis and suspected macrosomia, this element makes one think that the pathology might predispose to cesarean section. In terms of the size of the tumor, the biggest was found in pregnancies at term (3 over 7 with size > 5 cm), probably due to the longer time given to the neoplasm to grow. Considering that only one case of IUGR was found in a relatively small lesion (3 cm) and that the only case associated with maternal metastasis was found in lesions of minor diameters, the volume of the neoplasm doesn’t seem to be related to fetal or maternal outcome. The biggest choangiocarcinoma had a normal fetal and maternal outcome. The low malignancy is evidenced by the rapid decrease of the beta-HCG observed in the majority of cases.

It is not possible to define the recurrence of the pathology as the literature present is scarce.

Clinically silent, it usually appears as an incidental finding at 20 weeks or third-trimester ultrasound mostly as an anechoic or unevenly echogenic well-demarcated nodule, sometimes mimicking a placental-isolated infarct. The natural history of the disease is unknown, considering the paucity of the cases and the lack of a management protocol. Among the reported cases, there were no cases of chorangiocarcinoma showing evidence of tumor spread at delivery. This suggests a benign clinical behavior. However, chorangiocarcinoma may rarely have a malignant course, as demonstrated in one of the cases described in the literature [15]. For this reason, we suggest careful examination and follow-up of both the mothers and the babies.

## 5. Conclusions

In the past, the histological identity of chorangiocarcinoma has been questioned. With this systematic review, we tried to define the histological and clinical entity of this pathology. Its clinical course, in most cases benign, has probably underestimated its real incidence. Only greater knowledge of its histology and awareness of its clinical course will allow the future to evaluate the real prevalence of the disease and to stratify the risk of affected patients.

## Figures and Tables

**Table 1:** JBI Critical Appraisal Checklist for Reviews.

Author, Year	Study Type	D1	D2	D3	D4	D5	D6	D7	D8
Jauniaux et al. (1988) [13] Placenta	Case report	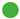	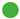	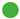	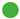	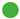	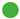	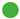	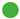
Trask C. et al. (1994) [9] Int J Gynecol Pathol	Case report	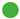	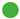	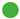	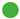	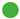	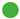	Not applicable	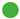
Ariel I. et al. (2009) [14] Int J Gynecol Pathol	Case report	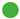	Not applicable	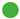	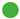	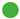	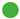	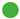	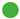
Guschmann M. et al. (2003) [8] Pathologe	Case report	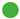	Unclear	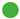	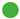	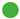	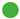	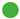	Unclear
Faes T. et al. (2012) [7] Placenta	Case report	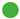	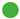	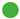	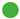	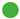	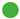	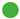	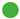
Huang B. et al. (2015) [15] Int J Clin Exp Med	Case report	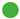	Not applicable	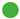	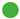	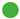	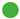	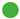	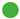
Garcìa-Molina F. et al. (2016) [16] Patalogìa	Case report	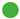	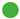	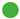	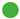	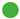	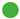	Not applicable	Unclear
Sagar N. et al. (2021) [17] Turk Patoloji Derg	Case report	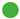	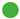	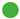	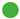	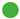	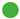	Not applicable	Unclear

JBI Critical Appraisal Checklist For Case Reports. D1. Were patients’ demographic characteristics clearly described? D2. Was the patient’s history clearly described and presented as a timeline? D3. Was the current clinical condition of the patient on presentation clearly described? D4. Were diagnostic tests or assessment methods and the results clearly described? D5. Was the intervention(s) or treatment procedure(s) clearly described? D6. Was the post-intervention clinical condition clearly described? D7. Were adverse events (harms) or unanticipated events identified and described? D8. Does the case report provide takeaway lessons?

**Table 2:** Reports of the literature.

Reference	Maternal Age	GA at Delivery (Weeks), Parity Singleton/Twin	Placental Weight (g)	Ultrasound Features	Macroscopic Examination Tumor Size	Microscopic Examination	Immunostaining	Maternal b-HCG (Baseline, Follow-Ups)	Fetal Assessment	Follow-Up	Maternal Outcome
Jauniaux E. et al., 1988 [13]	35	35 w, 3003 CS for shoulder presentation and vaginal bleeding Singleton	600	Not determined	Well-demarcated round nodule, multilobulated, limited by a white pseudocapsule 1.5 × 1.5 cm	Well-differentiated capillary pattern supported by chorionic stroma cells and fibrous tissue with an outer layer of syncytiotrophoblast and cytotrophoblast with nuclear atypia	-PAS-positive fibrin deposition in the area close to the tumor -hCG strongly positive in the trophoblastic layer and pseudocapsule -hPL positive to syncytium of the villi	-Not determined -Below detection 6 months after delivery	Normal	9 months	Normal
Trask C. et al., 1994 [9]	36	36 w, 1011 Spontaneous labor and delivery Twin BC/BA	250 (presenting twin placenta)	Not determined	Firm lesion similar to an infarct 3 × 2.5 cm	Stem villi with pronounced proliferation of villous stromal vessels, circumferential proliferation of malignant trophoblast protruding in the intervillous space (nuclear atypia, high mitotic rate)	-keratin (E1/AE3, Boehringer-Mannheim, Indianapolis, IN, U.S.A.) reactivity in the abnormal trophoblast -hCG positive -hPL weak and focal at the malignant trophoblast, strong in the normal syncytiotrophoblast	-Not determined -698 mIU/mL 7 days after delivery -Below detection 29 days and 3 months after delivery	Normal	7 days 29 days 3 months	Normal
Ariel I. et al., 2009 [14]	23	37 w, 0000 Spontaneous labor and CS for condylomata Singleton	678	Thick lesion of 5–6 cm with hyperechogenic and hypoechogenic areas without hypervascularization	Well-demarcated mass with alternating red and yellowish tissue on cut sections 8 × 5 cm	Malignant epithelial tumor with central necrosis forming a complex branching structure within a chorangioma (mitotic rate >90%, pleomorphic nuclei)	-Panytokeratin positive -b-HCG positive -hsd3b1 focally positive -hPL weakly positive	-Consistent with GA at baseline -Below detection 1 month after delivery	Normal	1 month after delivery	Normal
Guschmann M. et al., 2003 [8]	31	34 w, 1001 CS for fetal distress and FGR Singleton	496	Not determined	Nodule with grey-yellow foci 3 cm	Villi surrounded by syncytiotrophoblast atypia and chorangiosis with adjacent areas of necrosis (nuclear atypia, mitoses)	-bHCG at the syncytiotrophoblast -hPL weak and prevalent at the intermediate trophoblast -VEGF, bFGF, Ang-1,2, PDGF expression at the trophoblast similar to normal villi	-Consistent with GA at baseline -Not determined	FGR	Not determined	Normal
Faes T. et al., 2012 [7]	36	40 w, 2002 Spontaneous labor and delivery Singleton	812	Nodule of 8 × 7 cm with hyperechogenic and hypoechogenic areas with hypervascularisation at the border. Supplying artery PI 0.87, RI 0.58, PSF 39.34 cm/s.	Firm reddish-brown tumor with a lobulated appearance on section with multiple small white nodules 8 × 7 cm	Dilated angiomatous vessels filled up with neoplastic cell proliferation and separated by fibrous septa containing numerous capillary-type blood vessels; extensive central necrosis with dystrophic calcification (nuclei pleomorphism, multinucleation, high mitotic rate).	-AE1/AE3 cytoplasmic positivity -p63 nuclear staining -inhibin-alfa focal positivity -b-HCG strong positivity at the margins -Ki-67 high proliferation index	-Not determined -Below detection 1 month after delivery	Normal	1 month after delivery	Normal
Huang B. et al., 2015 [15]	27	39 w, 0000 Spontaneous labor and CS for suspicious of Macrosomia Singleton	500	Not determined	Firm grayish yellow-white mass with the consistency of an infarct. 5 × 4.5 cm	Abnormal trophoblastic proliferation in conjunction with a chorangioma in the stroma of chorionic villi. Cells forming solid masses with massive central coagulation necrosis surrounded by three to six layers of epithelial tumor cells (high proliferation index, mitoses)	-strong intensity for hCG, PLAP, CK, CD31 (+) and CD34 (+) in the lesion. -Ki67 high proliferation index	-Consistent with GA at baseline -Increase at 6 weeks after delivery -Below detection after 3 cycles of chemotherapy	Normal	-6 weeks after delivery -3 months after delivery	Lung metastasis at 3 months after delivery
Garcìa-Molina F. et al. (2016) Patalogìa [16]	36	41 3013 Elective C section Twin BC/BA	587 (fused bichorial placenta)	Not determined	Small irregular whitish area	Abnormal trophoblastic proliferation, with cellular atypia and arborescent proliferation through stromal cells and abundant vessels (cellular mitoses, coagulation necrosis, pleomorfism)	-Positivity for bHCG, CK 8–18, PAN CK -vascular proliferation vimentina and CD-34 (+) -High Ki-67 at the area of trophoblastic proliferation	-Not determined -negative at one month after delivery	Normal	1 month after delivery	Normal
Sagar N. et al., 2021 [17]	29	30 w, 1021 pProm, Chorionamnionitis CS Singleton	Not determined	Small hypoechoic lesion in relation to the uterine fundus with maintained uterine contour	Grey-white nodule, solid-cystic at cut section with friable areas 5.5 × 4.5 × 3 cm	Multiple well-circumscribed cellular nests with central necrosis dispersed in a chorangiomatous stroma (mitoses, apoptotic bodies, multinucleation)	-AE1/AE3, PathnSitu, U.S.A. cytokeratin positivity -b-HCH positivity -PLAP and Ki-67	-Consistent with GA at baseline -Not determined	Normal	Not determined	Normal

Table Legend: GA: gestational age; C.S.: Cesarean section; Beta HCG: beta human chorionic gonadotropin; W: Weeks.

## Data Availability

The authors confirm that the data supporting the findings of this study are available within the article.
